# The neuritogenic and neuroprotective potential of senegenin against Aβ-induced neurotoxicity in PC 12 cells

**DOI:** 10.1186/s12906-016-1006-3

**Published:** 2016-01-23

**Authors:** Robert Jesky, Hailong Chen

**Affiliations:** Department of General Surgery–Integrated traditional Chinese and Western medicine, 1st Affiliated Hospital of Dalian Medical University, No. 222, Zhongshan Road, Xigang District, Dalian, 116011 China

**Keywords:** Neuritogenesis, Neuroprotective, Nootropic, Cytotoxicity, PC 12 cells, Senegenin

## Abstract

**Background:**

Improved therapeutics aimed at ameliorating the devastating effects of neurodegenerative diseases, such as Alzheimer’s disease (AD), are pertinent to help attenuate their growing prevalence worldwide. One promising avenue for such therapeutics lies in botanical medicines that have been efficaciously employed in the likes of traditional medicine doctrines for millennium. Integral to this approach is the necessity of neuritogenesis and/or neuroprotection to counterbalance the deleterious effects of amyloid-β (Aβ) proteins. Senegenin, a principle saponin of *Polygala tenuifolia* Willd., which has empirically shown to improve cognition and intelligence, was chosen to evaluate its cytoprotective potential and possible neuritogenic and neuroprotective effects.

**Methods:**

The purpose of the present study was then to analyze morphological changes in neurite development and altered protein expression of two proteins requisite to neuritogenesis, growth associated protein 43 (Gap-43) and microtubule-associated protein 2 (MAP2) in PC 12 cells. Neuritogenic analysis was conducted with immunofluorescence after incubation with Aβ _(25–35)_ peptide, and to deduce information on cell viability and mitochondrial functionality MTT (3,(4,5-dimethylthiazol-2-yl)2,5-diphenyltetrazolium bromide) was employed.

**Results:**

This study found that cells pre-incubated with senegenin for 24 h (40 μg and 20 μg/ml) before introducing Aβ attenuated Aβ-cytotoxicity, and significantly increased cell viability by 23 % and 34 % (*P* < 0.001), respectively. In neurite outgrowth experiments, Aβ was compared to NGF positive control and senegenin treated groups which showed a drastic decrease in the quantity, average length and maximum length of neurites (*P* < 0.001). At concentrations of 1 μg/ml (*P* < 0.01) and 5 μg/ml (*P* < 0.05) senegenin triggered neuritogenesis with significant increases in total neurite number, average length and maximum length. This was additionally shown through the augmented expression of MAP2 and Gap-43.

**Conclusions:**

These results suggest that senegenin possesses cytoprotective properties, can moderate neurite outgrowth and augment MAP2 and Gap-43, thus suggesting a potential therapeutic value for *Polygala tenuifolia* in neurodegenerative disorders.

## Background

A major factor precipitating the pathogenesis of neurodegenerative illnesses such as Alzheimer’s and Parkinson’s disease is misfolded proteins, particularly amyloids. Neurons are highly susceptible to the deleterious effects of misfolded proteins which are prone to aggregate when large accumulations arise [1]. The pernicious effects of amyloid-β (Aβ) constitute one of the hallmarks of Alzheimer’s disease (AD). Alzheimer’s disease, a progressive neurodegenerative illness, characteristically results in severe cognitive deficits. The progressive cognitive decline associated with AD is attributable to dystrophic neurites and neuronal atrophy, which are the corollaries of extracellular amyloid-β peptide (Aβ) deposition and the intracellular deposition of neurofibrillary tangles (NFT) [2]; the latter of which is comprised of hyperphosphorylated tau proteins, and posited to be the resultant effect of the toxic cascade initiated by Aβ. The neurotoxic properties of Aβ have thus been formulated in an amyloid cascade hypothesis which posits that aggregates of Aβ 42 accumulate into senile plaques, either triggering complement factors and cytokines or directly leading to synaptic degeneration bypassing the activation of complement factors and cytokines, continually proceeding to alter ionic homeostasis and kinase activity. Ultimately, progressive neuritic dysfunction, cell death and neurotransmitter deficits result [3]. Current postulates, however, now strongly support the notion that the initial memory loss seen in AD directly correlates to oligomeric forms of Aβ, which precipitate synaptic deterioration [4–7]. Therefore, with the incidence of AD, the most common form of dementia, steadily on the rise, and a global prevalence predicted to affect over 80 million people by 2040 [8], the development of efficacious therapeutics aimed at abating AD pathogenesis, most notably neuritic and synaptic regeneration, is a necessity. Deciphering the complexities of AD stands at the forefront of neuroscience, with extensive research efforts demonstrating that Aβ is neuro-and synapto-toxic in both in vitro and in vivo experiments. Various research models have employed the use of the Aβ _(25–35)_ peptide, which retains the toxic properties of the full length peptide (39–43), to test the pathogenic mechanisms of Aβ, making it a desirable model for testing AD, and thus chosen for the present study to examine its toxicity in a neuronal cell line.

The PC 12 cell line, originally obtained from a transplantable rat pheochromocytoma [9], has become a widely used neuronal model to test neurotoxins implicated in neurodegenerative disorders, notably Aβ. It has additionally proven to be advantageous in expanding current understandings of what cellular proteins/mechanisms drive neurite growth. In the presence of neurotrophic factors, namely nerve growth factor (NGF), PC 12 cells cease division, and differentiate into sympathetic-like neurons producing long neurites [9, 10]. Upon differentiation PC 12 cells begin to express several integral proteins responsible for neurite growth [11, 12]. Growth associated protein 43 (Gap-43), a major polypeptide localized in the growth cone [13], is required for growing neuronal processes during development and regeneration [14]. Induction of Gap-43 by NGF has been reported to take place within 24 h, and is a prerequisite of neurite outgrowth in PC 12 cells [15]. Gap-43 is thus an essential protein imperative to the various stages underlying neural plasticity with a strong correlation to the growth phase of neurons, making it a good indicator of regeneration. Moreover, plasticity alterations in the brain evidently require the polymerization of microtubules, which play a paramount role in neuronal growth processes [16, 17], and intracellular signaling, with an integral relationship to MAPs that are mobilized during dentritic and axonal development. Microtubule-associated proteins (MAPs) regulate tubulin polymerization and are cardinal in microtubule assembly [11]. MAP2 shares a reciprocal relationship with mitogen-activated protein kinases (MAPKs) by being a cognate substrate. MAPKs NGF-stimulated activities and its association with phosphorylation states putative to neuritogenesis [18, 19] illustrate that MAPK is a pertinent cellular component of MAP2 function. Induction of neurite growth in PC 12 cells appears to be dependent upon the activation of MAPK, in that the inhibition of MAP kinase completely blocks NGF-induced neurite growth. Similarly, brief activation of MAP kinase by other growth factors has been shown to result in reduced PC 12 differentiation [20]. Not surprisingly, PC 12 neurite outgrowth requires similar intracellular mechanisms as hippocampal neurons, for instance, those that drive microtubule assembly and trigger MAPs [17, 21]. Another indispensable component of neuronal functionality, and imperative for neuritic regeneration, is the requisite synaptic terminal and its vesicles, both of which are additionally expressed PC 12 cells [22].

A novel approach to tackling the complexities of neurodegenerative disorders lies in the plasticity of the brain, which undergoes continued functional and structural alterations throughout life [23, 24]. Extensive research has shown that long after postnatal development the brain continues to birth new neurons (neurogenesis), primarily in the dentate gyrus of the hippocampus [25, 26], a structure well known for memory processing. Influential factors that contribute to neural plasticity are continuously being elucidated, with paramount importance being the natural, endogenous structural adaptations induced through neurotrophins. To date, various neurotrophins, including NGF, have proven to be efficacious in increasing synaptic efficacy, promoting neuritogenesis, synaptogenesis, and neuronal survival [27–30]. The use of neurotrophins as therapeutic agents, however, is limited since due to their molecular structure they don’t readily cross the blood brain barrier [31–34]. Therefore, exogenous substances that either trigger neurotrophins or independently contribute directly or indirectly through intracellular signaling mechanisms to neurito-and-synaptogenesis are likely to be an essential element of future therapeutics that halt neurodegeneration. Numerous plant sources have congruently been shown to possess nootropic activity and promote functional and structural changes in various cell types.

**Fig. 1:**
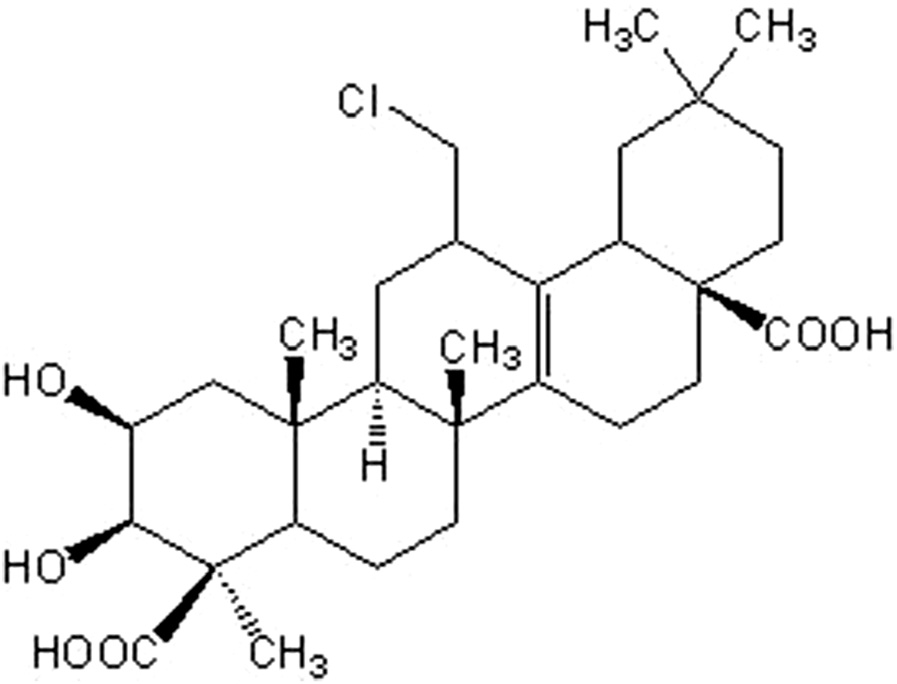
Chemical structure of Senegenin

The root of *Polygala tenuifolia* Willd. has been used for more than a millennia in traditional Chinese medicine for the treatment of memory loss associated with aging, forgetfulness and amnesia. Several of the most commonly used empirical traditional Chinese medicine formulas aimed at improving cognition contain *Polygala tenuifolia* [35]. Biochemical analysis has ascertained that the active components of P. *tenuifolia* are primarily saponins that are derivatives of presenegenin. Current research efforts directed towards elucidating the therapeutics of P. *tenuifolia* have shown that it possesses both neuroprotective and nootropic activity, exemplified in its ability to effectively attenuate scopolamine-induced memory impairments [36, 37], decrease Aβ secretion [38, 39], up regulate neurotransmitters [40], and increase NGF secretion is cultured astrocytes [41]. In addition, P. *tenuifolia* has been shown to promote the proliferation of hippocampal stem cells and neurite outgrowth [42], demonstrating that it’s a promising agent in the amelioration of neurodegeneration. Therefore, the aim of this research was to explore the potential neuroprotective and neuritogenic properties of senegenin (Fig. 1), a component of P. *tenuifolia* root extracts, on Aβ _(25–35)_-induced cyto-and-neurito-toxicity in differentiated PC 12 cells.

## Methods

### Materials

Aβ _(25–35)_ peptide fragment, poly-D-lysine, and 3-(4,5-dimethylthioazol-2-yl) 2,5 diphenyltetrazolium bromide (MTT) were all obtained from Sigma Chemical Co., (St. Louis, USA). Dimethyl sulfoxide (DMSO) was purchased from AMRESCO, (Solon, OH, USA). Mouse β-NGF was purchased from PeproTech Asia, (Rehovot, Israel). All tissue culture agents were purchased from Thermo Scientific Hyclone (Utah, USA). *S*enegenin - CAS No.667438-01-9, > 98 %HPLC) was obtained from Mansite Pharmaceutical Co., LTD. (Chengdu, China).

### Cell culture

PC 12 cells were obtained from Shanghai Institute for Biological Science Cell Bank, CAS, (Shanghai, China) and maintained in high glucose Dulbecco’s Modified Eagle Medium (DMEM) supplemented with 5 % (v/v) heat-inactivated horse serum, 5 % (v/v) heat-inactivated fetal bovine serum, a mixture of 1 % penicillin/streptomycin and incubated in an atmosphere with 5 % CO2/ 95 % humidified air at 37 °C. Cells were kept in 25 cm^2^ cell culture flasks (Corning Incorporated, USA) and dissociated every 3–4 days. Prior to all treatments cells were plated on either poly-D-lysine-coated 96-well microplates (Corning Incorporated, USA) or 1.8 cm^2^ glass cover slips to facilitate PC 12 differentiation. PC 12 cell differentiation was induced with differentiating medium (DM) which was comprised of DMEM, 1 % donar equine serum, 1 % penicillin/streptomycin, and 100 ng/ml mouse β-NGF. Every 3–4 days half of the differentiating medium was changed and replaced with fresh DM. All cells tested in this experiment underwent differentiation prior to evaluation.

### Cell viability assay

Cell viability was determined by MTT (3,(4,5-dimethylthiazol-2-yl)2,5-diphenyltetrazolium bromide) to provide information on cell viability and mitochondrial functionality. Cells were seeded at a density of 1 × 10^4^ in 100 μl DM (NGF 100 ng/ml) onto 96-well poly-D-lysine-coated plates for 4 days to facilitate neurite growth. After 4 days, cells were pre-treated with or without senegenin for 24 h, followed by incubation with Aβ _(25–35)_ peptide for another 24 h. Prior to use, Aβ _(25–35)_ was incubated at 37 °C for 4 days to allow aggregation. After incubating MTT (20 μl) for 4 h at 37 °C the formazan crystals were lysed in 150 μl dimethyl sulfoxide (DMSO), and the microplates were shaken vigorously to ensure complete solubilization. The optical density was 584 nm and read/determined in a Miltiskan MK 3 reader (Thermo Lab Systems Beverly, MA, USA). All values were presented as means ± SEM of numbers obtained from 6 wells, from 3 separate experiments, and cell viability (%) was expressed as a percentage relative to the NGF-treated control cells.

### PC12 neurite outgrowth experiments

Coated 18 × 18 mm coverslips pretreated with poly-D-lysine were placed into six-well culture plates and PC 12 cells were then plated at a density of 1.4–1.6 × 10^4^ in 1.5 ml cell suspension per well in the presence of DM to allow for differentiation. The plates were maintained in a homeostatic environment (see above) and half of the DM was changed every 3–4 days. Upon 7 days of differentiation, Aβ (10 μM) was added for 4 days to measure the effects of neuritic degeneration. Subsequently, 4 days after Aβ treatment the medium was removed and replaced with fresh medium containing NGF with or without various concentrations of senegenin to measure potential neurite regeneration. Moreover, the application of neurite specific antibodies (GAP-43 (1:100), MAP2 (1:50)), were used as markers of neurite growth formation, respectively. For this study, neurites were defined as processes nothing less than 10 μm or equal to the diameter of the cell body. Morphological observation of neurite outgrowth was characterized by three different parameters: average neurite length, maximum neurite length and neurite quantity. Neurite outgrowth parameters were quantified by visual counting and manual tracing, and acquired through confocal microscopy.

### Immunfluorescence

Cells were plated at a density of 1.4–1.6 × 10^4^ on poly-D-lysine-coated 18 × 18 mm coverslips in six-well culture plates (1.5 ml cell suspension per well). Differentiated cells were fixed in paraformaldehyde for 15 min at room temperature, succeeded by 10 min fixation with ice cold acetone at −20 °C, followed by several rinses with PBS. Non-specific binding in 5 % goat serum and 0.2 % Triton X-100 (BioSource Invitrogen, Carlsbad, CA, USA) preceded incubation with antibodies against Gap-43, and MAP2 (Neomarkers, Fremont, CA, USA) for 1 h at 37 °C. After a series of washes with PBS-0.05 % Triton X-100, a secondary antibody conjugated to FITC (ZyMed, San Fransisco, CA, USA), was applied and just prior to microscopic examination the cell nuclei were stained with 4′-6-diamidino-2-phenylindole (DAPI). All slide images were acquired by using a Nikon Ti 200 laser scanning confocal microscope.

### Statistical analysis

The data were expressed as means ± S.E.M. One way ANOVA followed by the LSD *post hoc* test was done for multiple group comparisons of the data collected. *P* values < 0.05 were considered statistically significant. Statistical analysis was performed using SPSS 17.0.

## Results

### The effects of senegenin on Aβ-induced cytotoxicity of PC 12 cells

**Fig. 2:**
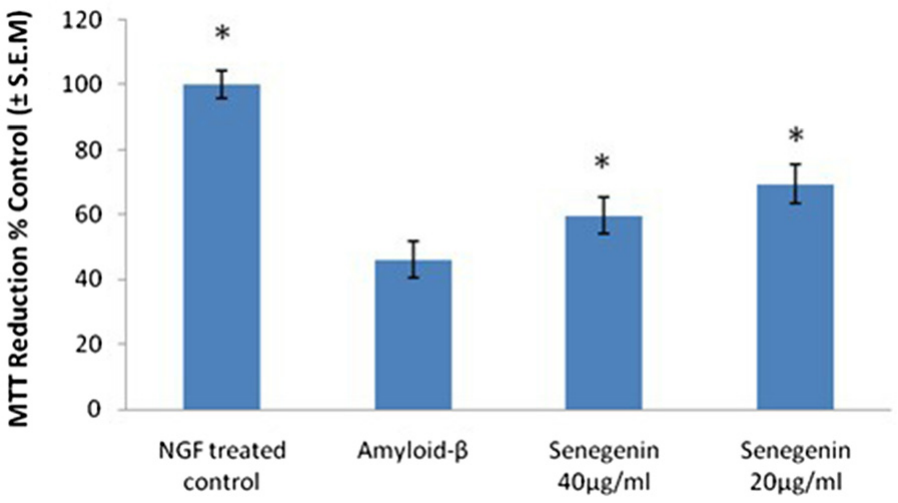
Effects of senegenin on MTT levels in Aβ-induced cytotoxicity in PC 12 cells. The cells were incubated for 24 h at 37 °C in the absence (Control & Aβ) or in the presence of senegenin (concentrations of 20 μg/ml & 40 μg/ml), and then incubated for 24 h followed by incubation with Aβ for 24 h. The data are the means ± S.E.M. of three separate experiments performed in triplicate. Cell viability (%) was expressed as a percentage relative to the NGF-treated control. *P < 0.001 vs. Amyloid-β treatment group

MTT was used to determine the effects of senegenin on the cell viability of PC 12 cells after Aβ-induced cytotoxicity. Following a 24 h incubation period with 10 μM Aβ _(25–35)_ cell viability was significantly reduced (Fig. 2) compared with control. Cells pre-incubated with senegenin for 24 h before introducing Aβ significantly increased cell viability, in dose-dependent manners as previously described [43]. When administered at concentrations of 40 μg/ml and 20 μg/ml senegenin significantly attenuated Aβ cytotoxicity (Fig. 2), increasing cell viability 23 % and 34 %, respectively. These results demonstrate the cytoprotective capabilities of senegenin against Aβ cytotoxicity. In addition, ginsenoside Rg1 attenuated cytotoxicity up to 21 % (data not shown). Comparatively, this shows that senegenin offers greater cellular protection than Rg1, a commonly employed derivative (ginsenoside) of *Panax ginseng* that has shown to effectively attenuate Aβ-induced cytotoxicity in numerous experiments [44–46].

### Morphological observation of senegenin neurite protection upon NGF removal

**Fig. 3:**
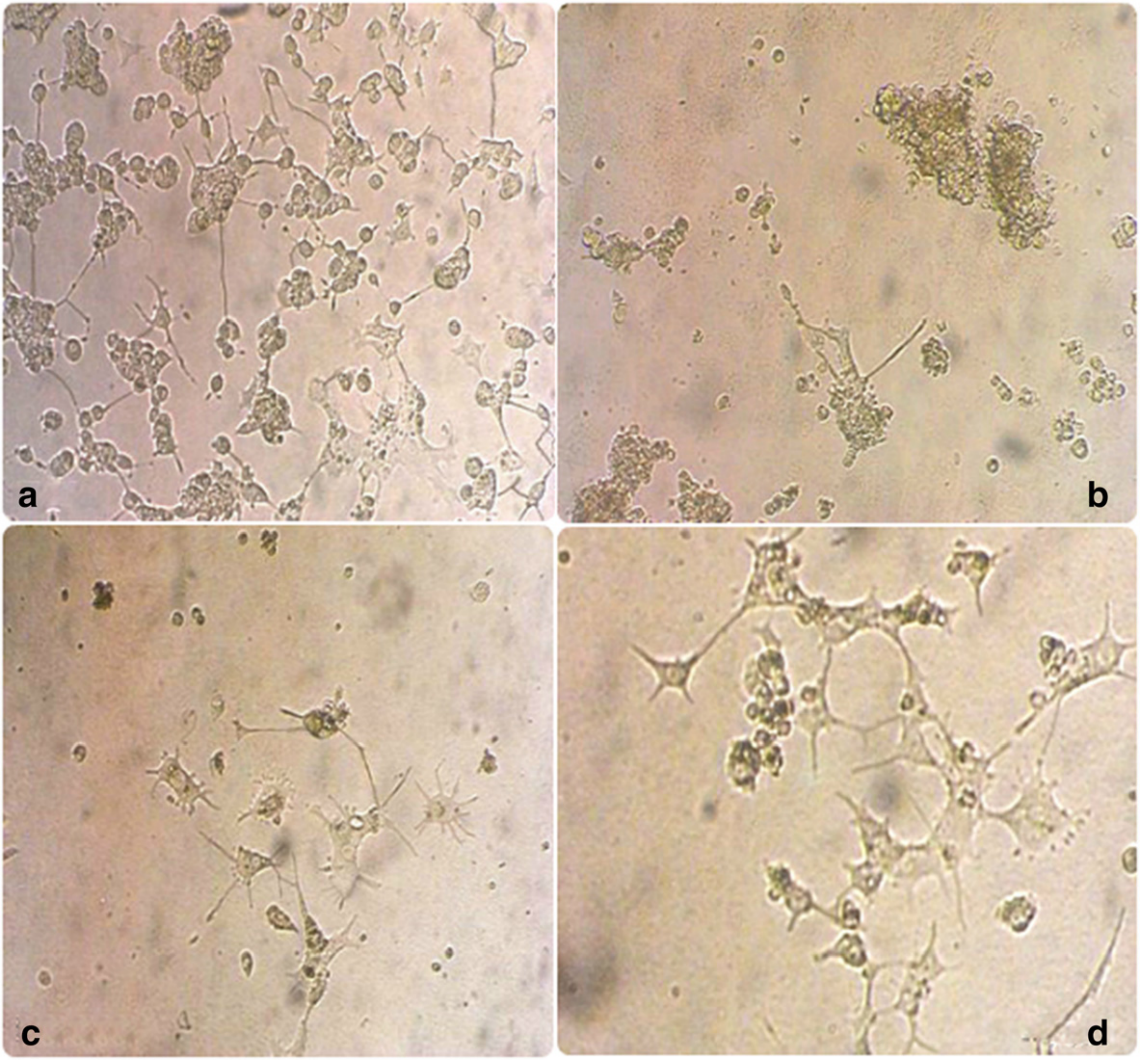
Morphological examination of PC 12 cells with or without senegenin. **a** represents PC 12 cells after 7 days incubation with DM (NGF 100 ng/ml). 48 h after the removal of DM, PC 12 cells loose neuritic processes and begin to proliferate (**b**). After the removal of DM, 5 μg/ml senegenin was added to the well. Morphological examination shows decreased axonal atrophy suggesting a neuroprotective effect (**c**). **d** illustrates retained neurite growth in the presence of 5 μg/ml senegenin 48 h after removal of DM

Observational analysis conducted by microscope confirmed that senegenin can protect neurites of differentiated PC 12 cells from the removal of NGF (reverting from DM back to culture medium). Cells grown for 10 days in DM showed long neuritic outgrowth (Fig. 3a); however, subsequent removal of NGF lead to retraction of neurites. 48 h after the removal of NGF differentiated PC 12 cells retrogressed to the proliferating stage with substantial cell clumping, and fragmented and atrophied neurites (Fig. 3b). Cells that were treated with senegenin (5 μg/ml) retained greater neurite distribution, length and number (Fig. 3c, d); thus, morphologically demonstrating the neuroprotective efficacy of senegenin on neurites.

### Neuritogenic efficacy of senegenin in Aβ-induced neuritic degeneration

**Fig. 4:**
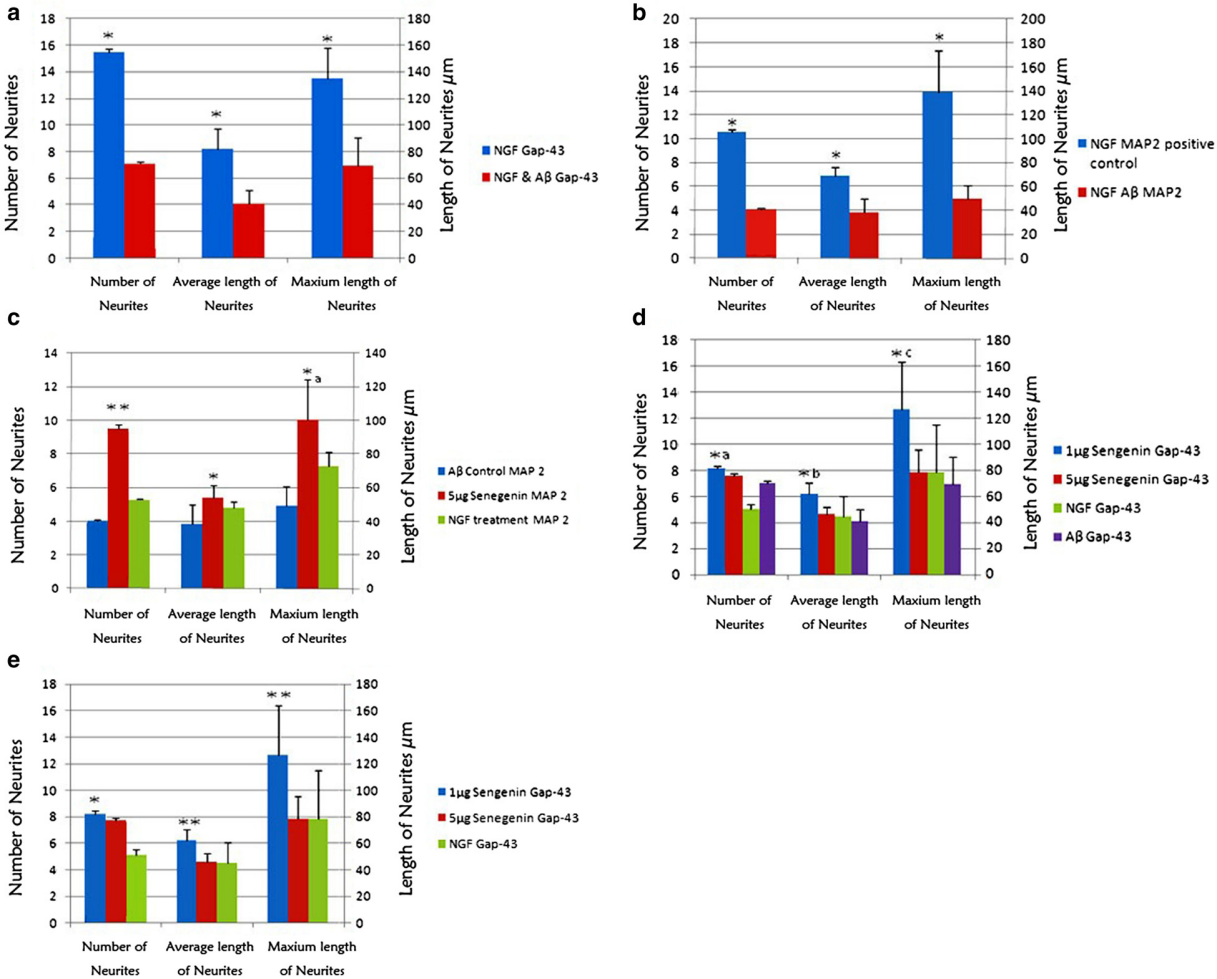
**a** The variance between the expression of Gap-43 positive neurites in NGF positive controlled growth and Aβ-induced toxicity. After 7 days of growth cells were treated with or without 10 μm Aβ _(25–35)_ for 4 days and then fixed and marked with Gap-43 and examined by immunofluorescence. In the absence of Aβ, neurites retain optimal growth and higher cell differentiation. *P < 0.001 vs. amyloid-β treatment group. **b** The variance between the expression of MAP2 positive neurites in NGF positive controlled growth and Aβ-induced toxicity following the same protocol as above (see **a**). In the absence of Aβ neurites retain optimal growth and higher cell differentiation. *P < 0.001 vs. amyloid-β treatment group. **c** The variance between the expression of MAP2 positive neurites in NGF and senegenin treated groups after Aβ-induced atrophy. After 7 days of growth cells were treated with or without 10 μm Aβ _(25–35)_ for 4 days. Subsequently, NGF and senegenin were introduced for an additional 4 days to incite regeneration. Next, the cells were fixed and marked with MAP2 and examined by immunofluorescence. In the presence of senegenin neurites expressed a higher protein level and a more significant outgrowth. **P < 0.01 vs. amyloid-β & NGF treatment groups. *P < 0.05 vs. amyloid-β treatment group. *^a^ *P* < 0.01 vs. amyloid-β treatment group. **d** The effects of senegenin on neuritogenesis after Aβ_(25–35)_-induced atrophy. PC 12 cells were differentiated for 7 days in the presence of NGF and were then treated with or without Aβ. 4 days after incubation with Aβ cells were treated with senegenin at concentrations of 1 μg & 5 μg/ml or NGF (100 ng/ml). Following an additional 4 days of treatment, the cells were then fixed and immunostained for phosphorylated Gap-43 as a neurite marker. The quantity, average length and maximum lengths were measured and compared. *^a^ *P* < 0.05 vs. NGF treatment group. *^b^ *P* < 0.05 vs. amyloid-β, NGF and 5 μg treatment groups. *^c^ *P* < 0.01 vs. amyloid-β, NGF and 1 μg treatment groups. **e** The differences in the efficacy of drug treatments aimed at neuritogenesis were measured following the protocol mentioned above, with 1 μg/ml evincing a marked effect. *P < 0.05 vs. NGF treatment. * *P < 0.05 vs. 5 μg senegenin and NGF treatments

**Fig. 5:**
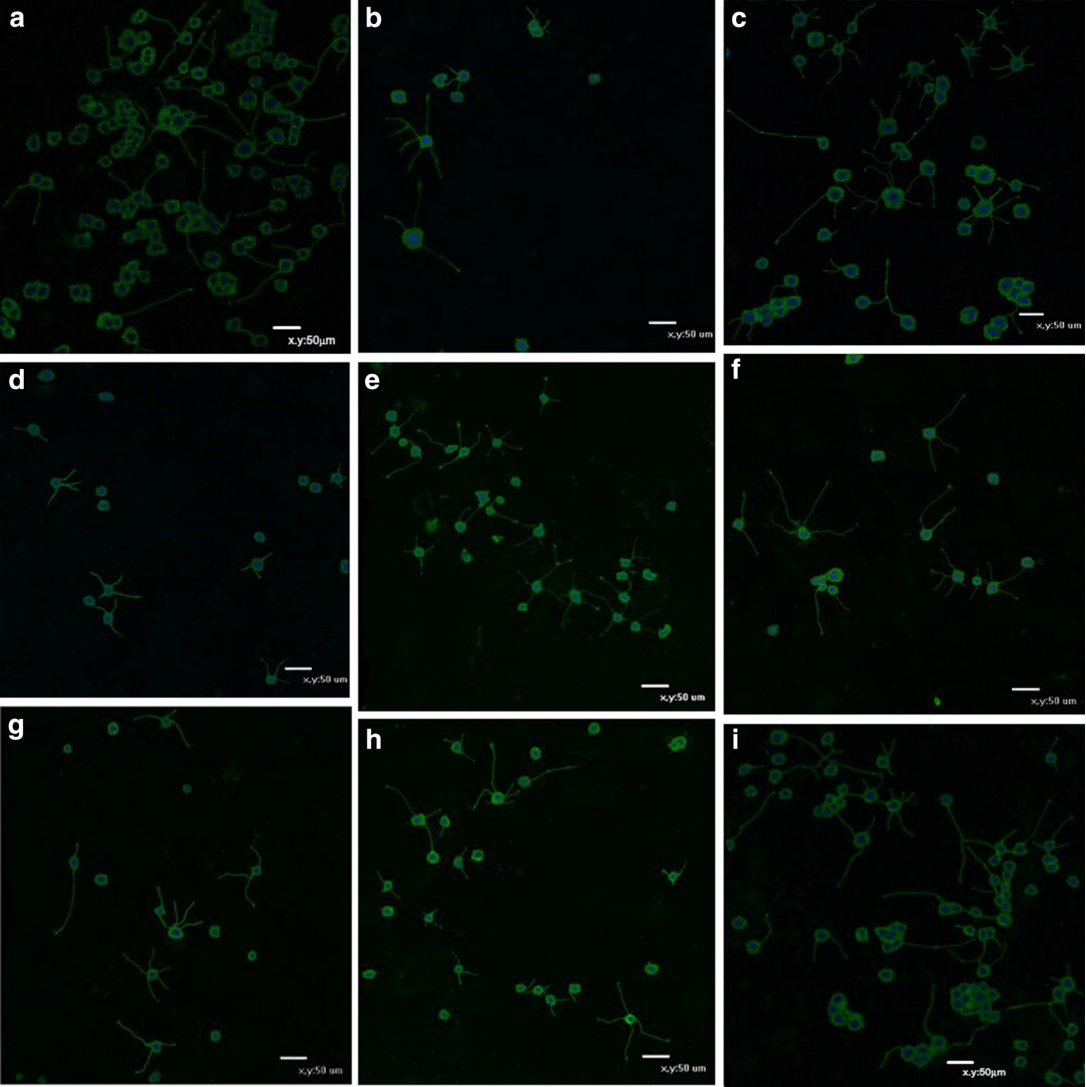
**a-i** displaying the effects of NGF, Aβ-induced neurite atrophy and neuritogenic outcomes of senegenin. **a** PC 12cells after 7 days of NGF-induced neurite outgrowth with positive expression of Gap-43. **b** Aβ-induced axonal atrophy and cell loss with additional reduction in Gap-43 expression. PC 12 cells were differentiated in DM for 7 days, thereafter Aβ was administered for 4 days. **c** PC 12 cells immunostained with MAP2 7 days after NGF-induced differentiation. **d** PC 12 cells were differentiated in DM for 7 days with subsequent incubation in the presence of Aβ for 4 days, then immunostained with MAP2 as a neurite marker. **e** Neuritogenic effect of senegenin (5 μg/ml) after 4 days of Aβ-induced atrophy. PC 12 cells show increased neurite outgrowth and MAP2 expression. **f** Neuritogenic effect of senegenin (5 μg/ml) after 4 days of Aβ-induced atrophy. PC 12 cells show increased neurite outgrowth and Gap-43 expression. **g** Neuritogenic effect of NGF (100 ng/ml) after 4 days of Aβ-induced atrophy. PC 12 cells show little neurite outgrowth w/ MAP2 expression. **h** Neuritogenic effect of NGF (100 ng/ml) after 4 days of Aβ-induced atrophy. PC 12 cells show moderate neurite outgrowth w/ Gap-43 expression. **i** Neuritogenic outcome in the presence of senegenin (1 μg/ml) after 4 days of Aβ-induced atrophy. PC 12 cells show increased neurite outgrowth w/ Gap-43 expression

PC 12 cells were cultured in the presence of 100 ng/ml β-NGF for 7 days to allow for significant differentiation (neurite outgrowth). After 7 days, half of the medium was changed which included the introduction of 10 μm Aβ _(25–35)_ into the DM for 4 days. Following 4 days of incubation differentiated PC 12 cells were fixed and labeled with primary antibodies for Gap-43 and MAP2 as neurite growth markers. Aβ drastically decreased total Gap-43 neurite values (*P* < 0.001), quantity 50 % of control, average length 41 % of control, and maximum length 65 % of control, (see Figs. 4a and 5a-b). Moreover, Aβ reduced MAP2 neurite values (*P* < 0.001) quantity 38 % of NGF positive control, average length 56 % of control and maximum length 35 % of control (see Figs. 4b and 5c-d), showing that Aβ _(25–35)_ induced significant neuritic atrophy.

Thereafter, the neuritogenic effect of senegenin in PC 12 cells was explored. PC 12 cells were grown in DM for 7 days, after which Aβ _(25–35)_ was introduced into the medium for an additional 4 days. Next, the medium was removed and replaced with fresh medium (DM) with or without (only NGF100 ng/ml) the presence of senegenin (1 μg & 5 μg/ml). The doses were chosen as a mean variance in reference to similar experimental results [47]. After 4 days treatment the cells were fixed and immunostained with Gap-43 and MAP2 as neuritic markers. The total quantity, average length, and maximum lengths were measured against Aβ _(25–35)_ control with marked decreases in Aβ-treated cells (see Figs. 4c-e and 5b, d). Treatment with senegenin significantly increased neurite outgrowth/regeneration in PC 12 cells in a concentration-dependent manner (*P* ≤ 0.01) Table 1. At concentrations exceeding 10 μg/ml senegenin did not significantly increase neuritogenesis (data not shown). Whereas, at concentrations of 1 μg/ml (*P* < 0.01) and 5 μg/ml (*P* < 0.05) senegenin triggered neuritogenesis showing marked increases in total neurite number, average length and maximum length (see Figs. 4c-d and 5e-f, i). Notably, the use of NGF alone to trigger regeneration after administration of Aβ did not significantly regenerate neurites compared to senegenin treatment or Aβ control (Figs. 4c-e, 5g, h). The use of NGF has shown to induce neurite growth in cortical neurons and PC 12 cells so its neuritogenic efficacy was measured against dose-dependent concentrations of senegenin wherein, of the concentrations utilized, it showed that 1 μg/ml (Fig. 4e) was the most effectual (*P* < 0.05) (Table 1).

**Table 1:** The changes in neurite composition under the influence of Aβ-induced degeneration and drug treatments

Treatments & protein expression	Neurite quantity	Average length	Maximum length
NGF positive control w/ Gap-43	15.5 ± 2.38*	81.75 ± 15.33*	134.75 ± 22.91*
NGF positive control w/MAP2	10.5 ± 1.29*	68.75 ± 7.23*	139.0 ± 34.42*
Amyloid-β w/Gap-43	7.0 ± 1.83	41.0 ± 9.63	69.5 ± 21.25
NGF control w/MAP2	5.25 ± 0.957	48.0 ± 3.56	73.5 ± 8.18
Amyloid-β w/MAP2	4.0 ± 0.826	38.25 ± 11.44	49.25 ± 11.27
Senegenin 5 μg w/Gap-43	7.75 ± 1.71	46.5 ± 5.8	78.25 ± 17.35
Senegenin 5 μg w/MAP2	9.5 ± 2.38*	54.25 ± 7.04*	100.5 ± 24.15*
Senegenin 1 μg w/Gap-43	8.25 ± 1.71*	62.25 ± 8.06*	126.75 ± 36.86*
NGF treatment w/Gap-43	5.0 ± 3.56	45.0 ± 15.64	78.5 ± 36.74

Cells were grown for 7 days in the presence of DM, followed by 4 days incubation with Aβ _(25–35)_ peptide. The medium was then changed to include various concentrations of drugs for an additional 4 days to allow for neurite outgrowth. PC 12 cells were then fixed and labeled with primary antibodies

*P < 0.01 vs. Amyloid-β treatment group

## Discussion

Severe neuronal atrophy with neuritic degeneration is a common pathogenic characteristic of neurodegenerative disorders. The possibility of regeneration presents to be of cardinal significance in abating neurodegeneration. To date, in vivo and in vitro experiments have demonstrated that axonal, dendritic and synaptic regeneration are possible [42, 46, 48–50]. One postulate that facilitates the regeneration of neuronal structures is the intracellular signaling proteins attributable to neural plasticity; possibilities that may be innate to the mammalian brain, but are somewhat repressed in the adult brain. Although under the influence of nootropic agents and neurotrophins they may become reactivated leading to neurito-and-synaptogenesis.

The aim of this present study was to evaluate the cytoprotective and neuritogenic/regenerative potential of senegenin. Cell viability, a valuable indication of mitochondrial function and integral to cellular differentiation, signaling and growth [51] was chosen to test the neurotoxic properties of Aβ. Aβ _(25–35)_, a commonly employed neurotoxic peptide [52–55], decreased cell viability by ~60 %. Interestingly, senegenin, at dose-dependent concentrations (20 μg and 40 μg/ml), attenuated Aβ toxicity and significantly increased cell viability in differentiated PC 12 cells. Aβ-induced neurotoxicity is thought to be partially caused by the induction of apoptotic pathways [56] and proapoptotic factors such as reactive oxygen species (ROS), endoplasmic reticulum (ER) stress and nitric oxide (NO). Research has shown that Aβ targets the apoptosis signal-regulating kinase 1 (ASK1) – JNK pathway [57, 58] which, through the production of ROS, constitutes a major apoptotic signaling pathway. In PC 12 cells, Aβ-induced cell death results from ROS-mediated activation of ASK1, which is independent of the ER stress pathway [57]. Caspase-2 has also been implicated in the induction of Aβ-induced apoptotic cell death in several neuronal populations including PC 12 [58]. In addition, c-Jun N-terminal kinase (JNK), which is activated by Aβ, plays a crucial role in triggering Fas ligand death factors that in turn leads to the binding of Fas ligand to its receptor Fas, and subsequently to the activation of a caspase cascade [59]; thus supporting the implicated role of caspase-2 in apoptotic cell death. Based on the activation of the JNK pathway and the following cellular cascades induced by Aβ one possible mechanism by which senegenin exhibits its cytoprotective qualities is through inhibition of ASK1 and/or JNK pathways. Additional support for this postulate can be found in the prevention of apoptosis in NGF-deprived PC 12 cells through inhibition of JNK or c-Jun [59]. Although evidence supports this mechanism of action further research is required to delineate the potential relationship of senegenin and the JNK pathway.

The attributes of neural plasticity, which encompass neuritogenesis, are proving to be a promising avenue of exploration by elucidating the complexities of the neuronal framework; from which new therapeutic angles have begun to arise. From this perspective, the neuritogenic potential of senegenin was examined. Results showed that at dose-dependent concentrations (1 μg and 5 μg/ml) neurite outgrowth/regeneration was potentiated. The induction of neurite growth requires the coordinate expression of certain growth proteins such as Gap-43 and MAP2, both of which, in comparison to Aβ-treated groups, were up-regulated after introduction of senegenin. These results are in agreement with previous studies that demonstrated that during times of regeneration Gap-43 expression rises, and is essential to neurite formation where it is expressed in along the entire axon [60, 61], with the progression of neuritogenesis shifting the concentration of Gap-43 to more distal locations; notably, growth cones and synaptic regions [61]. Moreover, in the absence of Gap-43 via the introduction of anti-Gap-43 antibodies neuritogenesis is prevented [60]. Gap-43 is a phospho-protein and a major substrate of protein kinase C (PKC), which implies that Gap-43 regulation of growth cones relies on a correlative relationship with PKC-induced phosphorylation [62, 63]. Furthermore, diacylglycerols (DAGs) an endogenous activator of PKC has shown to induce neuritogenesis in ganglia explants [64]. In concurrence with the relationship between PKC, neurite outgrowth and Gap-43, one likely mechanism of action for the neuritogenic efficacy of senegenin is its possible stimulation of PKC, leading to increased phosphorylation of Gap-43, and therefore to the initiation of growth cone formation and enhanced neurite outgrowth. This postulate is concordant with Yankner et al. [65] results that demonstrated marked acceleration in initial neurite growth and greater sensitivity to NGF in PC 12 cells expressing higher levels of Gap-43. PKC has additionally been found to phosphorylate MAP2, which is associated with decreased cytoskeletal stability [66]. Conversely, dephosphorylation of MAP2 is predicted to have an inverse reaction, thereby promoting cytoskeletal stabilization. Such a change in phosphorylated states leads to the instability of microtubules, which in turn corresponds with neurite outgrowth in hippocampal neurons [67]. Previous studies have noted that MAPs are imperative to cytoskeletal regulation and neurite growth [17], especially in dendritic development where MAP2 is heavily enriched [68] and goes through various phosphorylated states [69] that are in part regulated by PKC [66, 70]. From this, some reasonable inferences can be made regarding senegenin’s mechanisms of action. One, since MAP2 goes through alternating states of phosphorylation inducing oscillations of stability within microtubules, which allows for neuritic formation and outgrowth the mobilization of MAPs must be integral to the growth stage in PC 12 cells. Two, while the activation of MAP2 is important for PC 12 differentiation there appears to be little correlation between NGF and PKC-induced phosphorylation of MAP2 [71], suggesting that the activation of MAP2 is not a result of NGF-induced activation of PKC. We then speculate that if senegenin triggers PKC and phosphorylation of MAP2 this would lead to a notable increase in neurite growth as demonstrated in this study. Correspondingly, the increased protein recognition of Gap-43 and MAP2 by immunfluorescence in differentiated PC 12 cells found in this experiment is concordant with the associative mechanisms of action and imperative roles that these proteins play in neuritic development and formation.

While it is worth noting that the neurite outgrowth elicited by senegenin may have been produced through potentiating NGF-induced outgrowth, results demonstrated that in the sole presence of senegenin (Fig. 3c-d) neurite outgrowth was maintained. We thus propose that the results elicited herein stemmed from senegenin’s effects on PKC and phosphorylation of MAP2 rather than potentiating NGF.

## Conclusion

Dysfunctional protein assembly precipitates the occurrence of neurodegenerative illnesses like AD through abnormal accumulation. A prototypical example is the neurotoxic peptide Aβ. Two of most significant corollaries of misfolded proteins are dystrophic neurites and neuronal atrophy. This implies that to effectively tackle neurodegenerative diseases desired therapeutics need to possess neuroprotective and neuritogenic capacities. A promising source of such potential therapeutics can be found in botanical medicines. P. *tenuifolia* has empirically shown to be efficacious in treating cognitive disorders, and was thus a fitting choice for exploration of neuroprotective and neuritogenic potential. The results generated in our study suggest that senegenin not only possesses cytoprotective, and neuritogenic properties, but that it can also augment the expression of cardinal growth proteins vital to neural plasticity. One possible mechanism by which senegenin exhibits its cytoprotective qualities is through inhibition of ASK1 and/or JNK pathways. While deducing the exact neuritogenic mechanisms of action went outside of our studies aim, concordant studies lead us to hypothesize that these results stemmed from senegenin’s activation of PKC and its phosphorylation of MAP2. Although these results are in agreement with other literature surrounding the neuritogenic and neuroprotective nature of P. *tenuifolia*, further research is required to elucidate senegenin’s exact neurogenic mechanisms and its full therapeutic range. All in all, these results continue to support and illuminate P. *tenuifolia’s* (i.e., senegenin) salubrious potential, and suggest it may be efficacious in the treatment of neurodegenerative disorders.

## Abbreviations

AD: Alzheimer’s disease
ASK1: apoptosis signal-regulating kinase 1
Aβ: amyloid-β
DAGs: diacylglycerols
DM: differentiating medium
DMEM: Dulbecco's Modified Eagle Medium
ER: endoplasmic reticulum
Gap-43: growth associated protein 43
JNK: c-Jun N-terminal kinase
MAP: microtubule-associated protein
MAPK: mitogen-activated protein kinases
MTT: (3,(4,5-dimethylthiazol-2-yl)2,5-diphenyltetrazolium bromide
NFT: neurofibrillary tangles
NGF: nerve growth factor
NO: nitric oxide
PC 12: pheochromocytoma cell line
PKC: protein kinase C
ROS: reactive oxygen species
